# P-1420. Tracking Shadows: Loss to Follow-up in Persons Living with HIV

**DOI:** 10.1093/ofid/ofae631.1595

**Published:** 2025-01-29

**Authors:** Mehak Bhatia, William Carrington, Lauren Touleyrou, Gretchen Newman

**Affiliations:** Wayne State UNiversity, Southgate, MI, Michigan; Wayne State University, Detroit, Michigan; Wayne State University, Detroit, Michigan; Wayne State University School of Medicine, Rochester Hills, MI

## Abstract

**Background:**

During the year 2020, HIV viral suppression was 89.4% in clients throughout the United States who receive care through the Ryan White HIV/AIDS program (RYHAP). This was a significant increase compared to 69.5% in 2010. Likewise, Wayne State University (WSU) Tolan Park Infectious Disease Clinic, which is also funded by RYHAP noted a 5% increase in the rate of viral suppression in 2020 compared to the previous year. Eighty-six percent of Tolan Park clients across all Ryan White programs were virally suppressed, eighty-seven percent of women and youth were virally suppressed. However, there was a decline in the overall patient pool in 2019. The goal of this study was to locate those lost-to-care as part of the WSU Tolan Park clinic.

**Methods:**

Clinicians reviewed a list of clients who were lost-to-care during 2019. These clients were individually called by nurse practitioners, patient advocates or early intervention staff (EIS) to re-engage in medical care. The quality improvement team then discussed feedback from staff and clients regarding barriers to medical care.

**Results:**

We contacted a total of 310 clients. Out of these, 40 (13%) were lost-to-care. Among the remaining clients, 95 (31%) were deceased, 64 (21%) were in-care at other clinics, 63 (20%) were currently in-care at Tolan park clinic, 41 (13%) had confirmed clinic appointments and 7 (2%) were out-of-state or incarcerated. Of the 40 clients returned to care at the Tolan Park clinic, route cause analysis revealed the main barriers to care including poor mental health, lack of transportation, schedule conflicts, relocation, poor financial resources, inadequate medication adherence and clinic fatigue.

**Conclusion:**

We report a significant number of patients who were lost-to-care at the WSU Tolan Park clinic. Our patients continue to experience poor mental health, poor medication adherence and several socioeconomic barriers that limit their access to continuity of care. Our results highlight the need for better tracking services and strategies to minimize the loss to follow-up in HIV clinics.

**Disclosures:**

**All Authors**: No reported disclosures

